# Bilateral ovarian metastatic squamous cell carcinoma arising from the uterine cervix and eluding the Mullerian mucosa

**DOI:** 10.1186/1746-1596-9-109

**Published:** 2014-06-04

**Authors:** Sunil Jaiman, Kameswari Surampudi, Sirisha Rao Gundabattula, Deepasha Garg

**Affiliations:** 1 Department of Anatomic & Perinatal Pathology and Cytology, Fernandez Hospital, Unit 3, Plot 769, Road No. 44, Jubilee Hills, Hyderabad 500033, India; 2 Department of Gynaecology, Fernandez Hospital, Unit 1, 4-1-1230, Bogulkunta, Hyderabad 500001, India

**Keywords:** Cervical cancer, Endophytic tumor, Bilateral ovarian metastasis, Ovarian squamous cell carcinoma

## Abstract

Bilateral ovarian metastasis from invasive squamous cell carcinoma of the cervix is a rare phenomenon with very few clinically significant cases described in the literature. Ovarian metastases when present are usually seen in association with bulky, advanced cervical squamous cell carcinomas with extensive involvement of the uterus.

We describe a 48 year old woman with clinically normal cervix whose hysterectomy and bilateral salpingo-oophorectomy performed for abnormal uterine bleeding, demonstrated high grade squamous intraepithelial lesion, moderately differentiated squamous cell carcinoma involving the deeper stroma of the uterus and bilateral ovarian metastases. Gross examination of the cervical canal and the uterine cavity did not show tumor while well circumscribed pearly white metastatic deposits were distinguished within the parenchyma of both the ovaries. Microscopy ascertained high grade squamous intraepithelial lesion with malignant cells invading the deeper cervical stroma and disseminating further as lymphovascular tumor emboli within the myometrium of the corpus uteri without involving the endometrium. Both the fallopian tubes exhibited lymphovascular tumor emboli without epithelial involvement while the parenchyma of both the ovaries showed metastatic deposits.

Although an isolated case of endophytic squamous cell carcinoma of the cervix with extensive lymphovascular invasion of the corpus uteri, both the fallopian tubes and bilateral ovarian deposits without involving either the endometrium or the tubal mucosa does not form a paradigm, this case brings to light the capricious behavior of cervical squamous cell carcinoma.

**Virtual Slides:**

The virtual slide(s) for this article can be found here: http://www.diagnosticpathology.diagnomx.eu/vs/1214687069122755

## Background

Ovarian metastases from squamous cell carcinoma (SCC) of the cervix are rare and reported in less than 1% of early stage cervical SCC [1]. The risk increases with advanced lesions and in most of these cases the lesions are often bulky. Nakanishi et al in their comparative study between SCC and adenocarcinoma of the uterine cervix reported that ovarian metastases were found in 1.3% and 6.3% of cases respectively. The incidence in those with adenocarcinoma was associated more closely with tumor size whereas it was more associated with clinical stage in SCC [2].

We present a case of endophytic SCC of the cervix with extensive lymphovascular tumor emboli disseminating within the stroma of the corpus uteri, the tuba uterina and perpetuating as parenchymal deposits within both the ovaries without involving either the endometrium or the tubal mucosa. This, to the best of our knowledge has not been published before.

## Case presentation

### Case report

A 48 year old P4L4 visited the Gynecology outpatient department with chief complaints of heavy vaginal bleeding for 10 days following an eight-month period of amenorrhea. Progestin therapy was initiated as there was no relief of menorrhagia with tranexamic acid. Apart from severe backache for which she was undergoing an orthopedic consult, there was no other significant contributory history.

**Figure 1:**
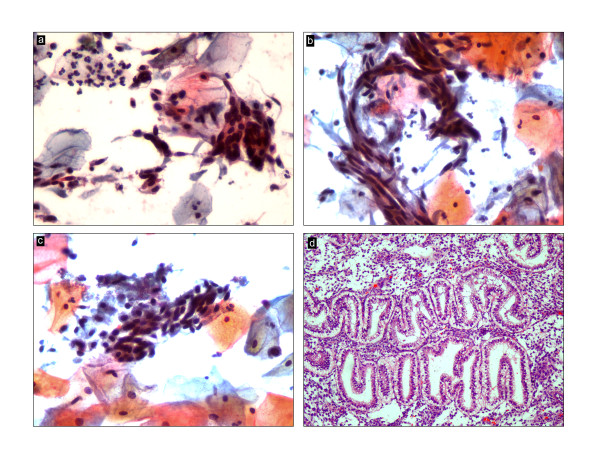
**Pap smear and endometrial biopsy. a, b** &**c**: High grade squamous intraepithelial lesion (Papanicolaou 400 x); **d**: Secretory endometrium (hematoxylin and eosin 100 x).

General physical and breast examination was unremarkable. The cervix and vagina appeared normal with no focal lesions and bimanual palpation disclosed an enlarged uterus corresponding to 14-16 weeks’ size. Ultrasonography revealed a bulky uterus with thickened endometrium. The ovaries were enlarged (right: 53 × 34 × 37 mm; left: 42×32×29 mm) but had a normal echotexture. At hysteroscopy, the endometrium appeared mildly hyperplastic and there was no abnormality in the cervical canal.Papanicolaou smear was initially reported as negative for intraepithelial lesions and malignancy (Figure 1a,b,c) while a simultaneously performed endometrial biopsy showed secretory endometrium, post ovulatory day 3 with concomitant exogenous hormone induced changes (Figure 1d). There was no evidence of endometritis, granulomas, hyperplasia, atypia or malignancy in the endometrial biopsy. Despite the availability of alternative treatment modalities such as oral progestins, endometrial ablation and Mirena levonorgestrel-intrauterine system, the patient opted for the removal of uterus. Consequently, total laparoscopic hysterectomy with bilateral salpingo-oophorectomy was performed.

### Pathologic findings

**Figure 2:**
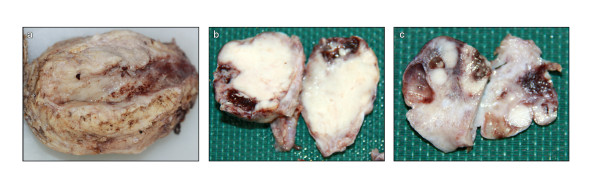
**Macroscopy of uterus and bilateral ovaries. a**: Bisected uterus; **b** &**c**: Cut surface right and left ovary

**Figure 3:**
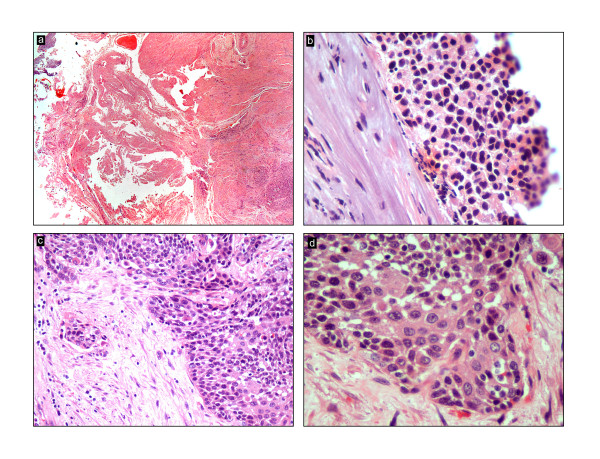
**Cervical intraepithelial neoplasia (CIN) stained with hematoxylin and eosin. a**: CIN with cautery artefact in underlying tissue (20 x); **b**: CIN (400 x); **c**: Stromal invasion (200 x); **d**: Stromal invasion (400 x)

**Figure 4:**
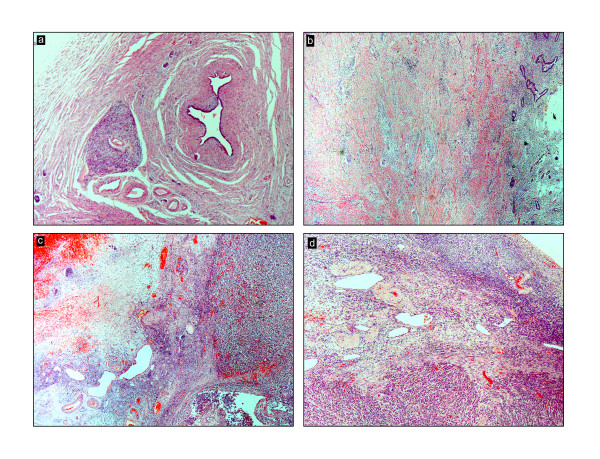
**Microscopic images of metastases stained with hematoxylin and eosin. a**: Left tubal stromal metastasis (20 x); **b**: Endomyometrial tumor emboli (20 x); **c**: Left ovarian metastasis (20 x); **d**: Right ovarian metastasis (40 x).

**Figure 5:**
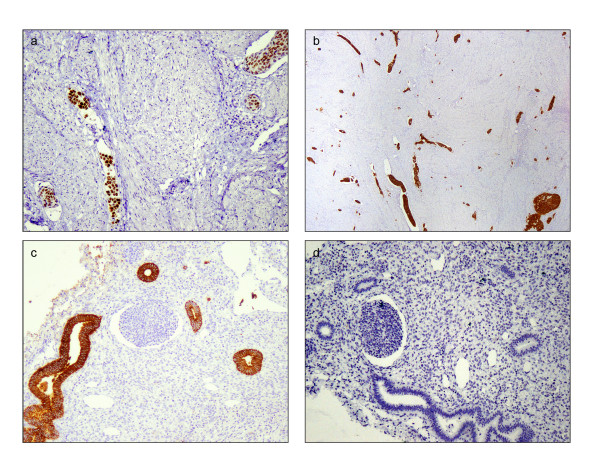
**Immunohistochemical staining of endomyometrium. a**: p63 positivity (100 x); **b**: High molecular weight cytokeratin CK5/6 positivity (20 x); **c**: CK7 negativity (100 x); **d**: CK20 negativity (100 x)

**Figure 6:**
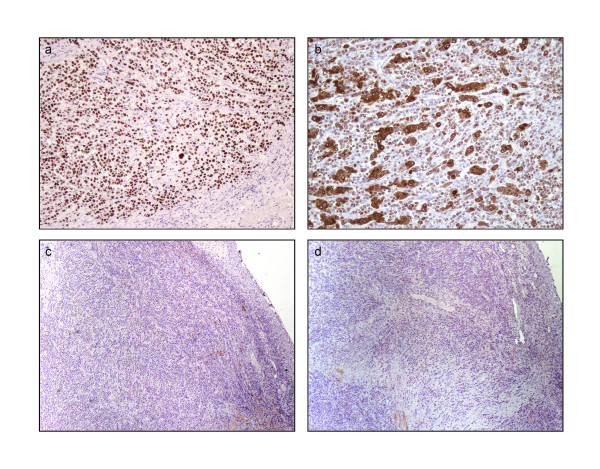
**Immunohistochemical staining of right ovary. a**: p63 positivity (100 x); **b**: High molecular weight cytokeratin CK5/6 positivity (100 x); **c**: CK7 negativity (40 x); **d**: CK20 negativity (40 x).

**Figure 7:**
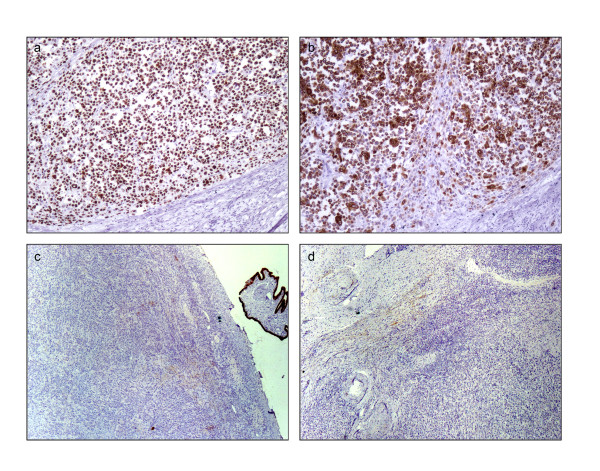
**Immunohistochemical staining of left ovary. a**: p63 positivity (100 x); **b**: High molecular weight cytokeratin CK5/6 positivity (100 x); **c**: CK7 negativity (40 x); **d**: CK20 negativity (40 x)

The uterus was received bisected (9.0 × 6.0 × 5.0 cm, 356.0 gm) with an essentially normal endometrial lining and thickened myometrium (Figure 2a). The exocervix (1.5 × 1.5 cm) had focally irregular mucosal surface. The endocervix (2.5 cm in length) was unremarkable. Small nodules varying in diameter from 0.5 to 1.0 cm were noticed near the fimbrial ends while the tubal lumina were patent without mucosal thickening. The right ovary had a convoluted external surface which upon sectioning demonstrated near complete replacement of the ovarian parenchyma by a grey white lesion (3.9 × 2.3 × 0.7 cm) (Figure 2b). There was no hemorrhage or necrosis. The left ovary had a convoluted external surface and the parenchyma showed two grey white well demarcated lesions (2.0 × 1.0 cm and 1.0 × 1.0 cm) and a single smooth walled cortical cyst (Figure 2c). After overnight fixation in 10% formalin and processing, the tissues were embedded in paraffin. Multiple 3 to 5 micron sections were cut and stained with hematoxylin and eosin. Immunohistochemical study was performed by Dako’s envision method.Microscopically cervical intraepithelial neoplasia grade 3 was detected over the surface epithelium while the deeper stroma exhibited islands of moderately differentiated SCC (Figure 3). The endometrium was weakly proliferative, completely uninvolved by tumor. Both the fallopian tubes showed hydrosalpinx and the lumina were devoid of tumor deposits (Figure 4a). There was ubiquitous presence of lymphovascular tumor emboli within the cervical stroma, myometrium and the tunica muscularis (Figure 4b). Both the ovaries showed well circumscribed nodular tumor deposits of moderately differentiated SCC (Figure 4c,d). Contiguous ovarian parenchyma showed normal stroma, corpora albicantes and thick walled vessels. The left ovary additionally showed a follicular cyst.The endophytic cervical lesion, the lymphovascular channels within the endomyometrium and the tubes, and the ovarian lesions demonstrated strong positivity to p63 and high molecular weight cytokeratin CK5/6 immunohistochemistry (IHC) markers (Figures 5a, b, 6a,b, 7a,b). Squamous differentiation is characterized by strong nuclear staining with p63 and cytoplasmic staining with CK5/6, and the positive staining of these markers corroborated with the neoplastic foci seen on hematoxylin and eosin sections. Significant negative markers included CK7/CK20 (Figures 5c,d, 6c,d, 7c,d), leukocyte common antigen, gross cystic disease fluid protein, CD117 and inhibin.

Morphology and IHC evaluation concluded that the bilateral ovarian metastases, ubiquitous tumor emboli in the lymphovascular channels of the fallopian tubes and endomyometrium, and the endophytic cervical lesion were all squamous and originated in the exocervical epithelium. Despite liberal sampling the mucosal involvement of the endometrium or the tubes could not be demonstrated. A review of the cervical cytology smears ascertained that neoplastic cells had been misinterpreted as reactive endocervical cells by a trainee neophyte pathologist.

## Discussion

Metastatic tumors to the ovary account for approximately 10 – 15% of ovarian malignancies [3] with the majority of the metastatic tumors arising from the genital tract. Cervical cancer is a very rare cause of ovarian metastasis [4] and the risk is more likely in advanced disease vis-à-vis early stage cervical cancer [5]. Most of the advanced cases described have bulky exophytic growths with extensive corpus uteri involvement. Metastases to ovaries occur in 0.5% of cases of SCC and 1.7% of cases of adenocarcinoma, so ovarian preservation at the time of surgery may incur a small risk of occult disease [5].

In a large autopsy series by Tabata et al [6] ovarian metastases were found in 104 out of 597 (17.4%) cases of SCC as opposed to 28.6% cases of adenocarcinoma while Toki et al [7] reported ovarian metastasis in only one case of 524 SCCs. Most of the ovarian metastases reported are microscopic, unilateral, confined to ovarian parenchyma and detected postoperatively [1,8-11]. Independent risk factors for ovarian metastasis include age, stage, non squamous histology, unaffected peripheral stromal thickness [12] and uterine corpus involvement [13].

Possible routes of spread to the ovary from cervical cancer include hematologic metastasis [14], or lymphatic drainage and transtubal drainage [6] and involvement of the corpus may potentiate these mechanisms. Reverse transcriptase in situ polymerase chain reaction for human papillomavirus ribonucleic acid is a reliable method to differentiate metastatic cervical carcinoma from either a new primary tumor or a metastasis from another cancer [15]. In the female genital tract, p63 is expressed in the basal and parabasal layers of mature cervical, vaginal, and vulval squamous epithelium, and is useful to establish the diagnosis of cervical squamous cell carcinoma [16].

Endophytic cervical squamous cell carcinoma with normal endocervical, endometrial and fallopian tube epithelia, extensive lymphovascular invasion of the entire genital tract and bilateral parenchymal involvement of the ovaries is an extremely rare presentation. Endophytic tumors may appear normal speculoscopically and colposcopically. The growth occurs in the cervical canal with direct infiltration into the wall causing diffuse enlargement and hardening of the cervix. The mucosal surface may be covered by normal epithelium, and the underlying malignant cells may escape detection by cytologic smear. These endophytic tumors may produce a barrel-shaped cervix, which has a diameter greater than 4 cm. Rectal examination can be helpful in such cases to palpate the enlarged uterine cervix and the role of MRI is usually complementary. Actual pathophysiological mechanisms leading to abnormal bleeding in carcinoma cervix are poorly understood, but are probably due to the presence and dilatation of abnormal surface vessels on the lesion [17].

Since the ovaries in our case demonstrated solid tumor bilaterally, primary solid ovarian neoplasms such as Brenner tumor, non cystic ovarian teratoma, dysgerminoma, granulosa cell tumor and lymphoma were excluded with the help of IHC markers. Apart from these, ovarian endometrioid adenocarcinoma resembling sex cord stromal tumor which demonstrates CK7 and epithelial membrane antigen positivity [18] was differentiated by negativity in our case. Endometrioid adenocarcinoma with squamous differentiation may show keratin granulomas over the surface of the ovary and if viable tumor cells are observed in the granulomas, these lesions should be regarded as conventional metastatic foci [19]. There were no keratin granulomas or peritoneal deposits in our case. Further the endophytic nature required differentiation from mesonephric adenocarcinoma with sarcomatous component [20]. Absence of sarcomatous component in the cervical biopsy helped exclude this lesion. Non-involvement of endometrium with invasive squamous cell carcinoma, along with demonstration of secretory changes and concomitant exogenous hormone induced features warranted that a FIGO grade 1 endometrioid adenocarcinoma and/or intestinal-type metaplasia be ruled out [21]. This was eliminated by CA 125, AB-PAS and CDX-2 IHC marker negativity within the endometrium while p63 and high molecular weight cytokeratin CK5/6 positivity in the cervix and metastatic ovarian deposits.

Differentiation between metastatic SCC from the cervix and primary SCC of the ovary usually has been aided by the knowledge of the presence of a cervical tumor. Before the diagnosis of a primary SCC of the ovary is made, the possibility of spread from a cervical tumor, even one that is occult should be considered unless overt features of primary neoplasia are immediately obvious. As most SCCs of the ovary arise in the background of a pre-existing neoplasm such as dermoid or endometriotic cyst, thorough sampling to identify such a component may be crucial in determining the primary nature of the neoplasm. Although the evidence strongly points to the ovarian tumor being metastatic when both organs have been involved by SCC, the rare association of SCC of the ovary with SCC in situ of the cervix leaves open the possibility of independent primary neoplasms in some cases [22].

## Conclusions

The ovarian involvement would have remained occult had our patient not opted for concomitant bilateral salpingo-oophorectomy with hysterectomy. Although the rarity of metastatic squamous cell carcinoma to the ovaries along with non-conventional spread of the lesion does not form a paradigm, its propensity to remain occult with catastrophic consequences suggests that there is a need to revisit the behavior of cervical SCC.

## Consent

Written informed consent was obtained from the patient for publication of this Case Report and any accompanying images. A copy of the written consent is available for review by the Editor-in-Chief of this journal.

## Abbreviations

SCC: Squamous cell carcinoma; IHC: Immunohistochemistry; FIGO: International Federation of Gynecology and Obstetrics; AB-PAS: Alcian blue-per iodic acid Schiff; CIN: Cervical intraepithelial neoplasia.

## Competing interests

The authors declare that they have no competing interests.

## Authors’ contributions

SJ carried out the histopathologic and immunohistochemical studies and collaborated in writing the manuscript. KS performed the surgery and provided intellectual contributions to the manuscript. SRG reviewed the manuscript critically for intellectual content. DG performed the initial Pap smear evaluation, grossed the hysterectomy specimen and wrote the introductory draft of the manuscript. All the authors have contributed significantly and are in agreement with the content of the manuscript.

## Authors’ information

SJ is the Head of the Department of Anatomic & Perinatal Pathology and Cytology, Fernandez Hospital. He is an Associate member of the Royal College of Pathologists, UK and a member of European Society of Pathology (ESP), Society for Pediatric Pathology (SPP), Indian Society of Perinatology and Reproductive Biology (ISOPARB), Indian Association of Pathologist & Microbiologists (IAPM), Indian Association of Pathologists & Microbiologists – Andhra Pradesh State Chapter, Society of Indian Academy of Medical Genetics, Delhi Medical Association Delhi state branch - Indian Medical Association and Founder Life Member of Association of Practicing Pathologists.

KS is the Head of the Department of Gynaecology, Fernandez Hospital. She is a Fellow of the Royal College of Obstetricians and Gynaecologists and a member of the British Society of Cervical Cytology and Pathology.
